# Physical Activity Is Associated with Reduced Implicit Learning but Enhanced Relational Memory and Executive Functioning in Young Adults

**DOI:** 10.1371/journal.pone.0162100

**Published:** 2016-09-01

**Authors:** Chelsea M. Stillman, Jennifer C. Watt, George A. Grove, Mariegold E. Wollam, Fatma Uyar, Maria Mataro, Neal J. Cohen, Darlene V. Howard, James H. Howard, Kirk I. Erickson

**Affiliations:** 1 Department of Psychiatry, University of Pittsburgh Medical School, Pittsburgh, PA, United States of America; 2 Department of Psychology, University of Pittsburgh, Pittsburgh, PA, United States of America; 3 Department of Psychiatry and Clinical Psychobiology, University of Barcelona, Barcelona, Spain; 4 Beckman Institute, University of Illinois at Urbana-Champaign, Urbana, IL, United States of America; 5 Department of Psychology, Georgetown University, Washington, D.C., United States of America; 6 Department of Psychology, The Catholic University of America, Washington, D.C., United States of America; University of Wisconsin-Milwaukee, UNITED STATES

## Abstract

Accumulating evidence suggests that physical activity improves explicit memory and executive cognitive functioning at the extreme ends of the lifespan (i.e., in older adults and children). However, it is unknown whether these associations hold for younger adults who are considered to be in their cognitive prime, or for implicit cognitive functions that do not depend on motor sequencing. Here we report the results of a study in which we examine the relationship between objectively measured physical activity and (1) explicit relational memory, (2) executive control, and (3) implicit probabilistic sequence learning in a sample of healthy, college-aged adults. The main finding was that physical activity was positively associated with explicit relational memory and executive control (replicating previous research), but negatively associated with implicit learning, particularly in females. These results raise the intriguing possibility that physical activity upregulates some cognitive processes, but downregulates others. Possible implications of this pattern of results for physical health and health habits are discussed.

## Introduction

It is widely accepted that physical activity improves and maintains physical health, but there is also evidence that it is associated with improved cognitive functioning. To date, most of the evidence for the salutary effects of physical activity on cognition comes from the field of cognitive aging. For example, more physically active older adults outperform their less physically active peers on a variety of cognitive tasks [1,2], and a greater amount of physical activity is associated with a lower risk of experiencing cognitive impairment over a 1–12 year period [3]. Randomized controlled trials of exercise also report improvements in cognitive functioning in older adults following aerobic exercise training compared to active control conditions (e.g., [4,5,6]). Meta-analyses of training studies have revealed that cognitive improvements following exercise training are particularly pronounced in the domains of memory and executive functioning [7,8]. Thus, there is evidence that physical activity improves cognitive functioning, and that there may be some specificity, such that it benefits some cognitive processes more than others (but see [9] for evidence to the contrary). Here, we expand the existing literature by examining, for the first time in the same study, the relationship between physical activity and explicit, as well as implicit cognitive functioning (specifically implicit probabilistic sequence learning). Further, we will do so in a sample of healthy younger adults rather than in the older adult samples typically tested in studies of physical activity and cognition.

The idea that physical activity may be particularly beneficial for memory and executive functioning in humans is perhaps not surprising considering what has long been known about the neural effects of exercise from animal models. Specifically, aerobic exercise increases neurogenesis in the hippocampus, a region central for learning and memory (e.g., [10]). For example, rodents given access to running wheels show enhanced proliferation and survival of newborn neurons in the dentate gyrus, a hippocampal subregion, as well as superior performance on hippocampal-dependent cognitive tasks (e.g., spatial memory, novel object recognition) compared to sedentary rodents (for review see [11]). In addition, exercise increases the proliferation of new capillary beds, increases expression of neurotrophic factors and neurotransmitters, and influences inflammatory cytokines (for review see [11]). Studies of aerobic exercise in rodents have therefore highlighted several mechanisms by which aerobic activity affects cognitive performance, suggesting that the effects of physical activity in humans are mediated by enhanced neural (particularly hippocampal) functioning. One important caveat of using animal models, however, is that the results cannot always be directly extrapolated to humans.

Neuroimaging studies of physical activity in humans complement the rodent literature. In older humans, a 12-month randomized controlled trial of aerobic exercise increased hippocampal volume, and these changes in hippocampal volume were associated with changes in spatial memory performance [4]. Others have since replicated the effects of exercise on hippocampal volume and cognitive performance [12,13] and have shown that exercise also affects the structure and functioning of other brain regions, such as the prefrontal and anterior cingulate cortices, regions supporting executive cognitive functions [14–16]. In addition to inducing structural brain changes, exercise improves the strength of functional communication between the hippocampus and prefrontal cortex, as well as amongst regions within large-scale brain networks [17,18]. The emerging evidence from fMRI studies of exercise in humans thus builds upon the animal work in that it suggests that physical activity not only impacts individual brain regions associated with specific cognitive functions—e.g., the hippocampus and prefrontal cortex—but that it may also have more global influences on brain communication and efficiency.

Despite the wealth of evidence that physical activity promotes both cognitive and brain health, there are still many open questions. For example, although studies of physical activity have included a variety of cognitive tasks measuring *explicit* cognitive functions (i.e., those that are goal-directed and subsequently verbalizable), few have included tasks assessing *implicit* functions (i.e., those that occur without awareness or intent and are not subsequently verbalizable). The primary goal of the present study was to examine whether the relationship between physical activity and cognition also extends to an implicit function, implicit probabilistic sequence learning (IL). IL is a cognitive process that involves acquiring probabilistic regularities from sequences of events. This type of learning is thought to underlie important everyday functions, such as developing new languages, adapting to new social and built environments, and forming new behavioral repertoires [19].

To measure IL in the laboratory, here we use the Triplets Learning Task (TLT) [20]. The TLT measures implicit probabilistic sequence learning that occurs independently of *motor* sequencing. Importantly, the more perceptual form of IL measured by the TLT differs from motor-based IL (the type measured in traditional sequence learning paradigms) because it requires participants to extract subtle sequential regularities from the environment independently of the influences of motor fluency [21]. Thus, IL in the TLT does not involve learning a motor sequence, and is not confounded by individual (or group) differences in motor capacity. However, the TLT shares several key qualities with motor-based sequence learning tasks, such as the fact that learning is sequential and (in most cases) implicit. Thus, we use “IL” when referring to both variations of implicit sequence learning generally, and we will call on evidence from both the motor and perceptual IL literatures to frame and interpret the present results. However, given the differences outlined above, we will highlight when the tasks employed across studies differ in terms of their motor demands and later speculate on if/when this matters in terms of the effects observed.

IL (both motor and perceptual) is thought to proceed in several stages, which can broadly be divided into the training or “acquisition” phase, and the offline or “consolidation” phase [22]. We focus on the acquisition phase in the present study. Within the contemporary IL literature, the acquisition phase is often further subdivided into an initial, early learning phase in which associations are being rapidly formed, and a later learning phase in which the probabilistic associations become more automatized [20,23,24]. This distinction may be critical as there is growing evidence that different neural systems may underlie the different phases of acquisition (e.g., [23]).

There is reason to expect that IL might be sensitive to individual differences in physical activity. This is because functional magnetic imaging (fMRI) studies have demonstrated that, in initial phases of both perceptual and motor-based IL, participants recruit the caudate nucleus, as well as the hippocampus, a region that, as mentioned above, is known to be particularly sensitive to exercise training [23,25–29]. In addition, the integrity of white matter tracts connecting the hippocampus and caudate to the dorsolateral prefrontal cortex (DLPFC) is associated with better motor-based IL performance in the early and late phases of training, respectively [30]. Thus, both the hippocampus and DLPFC, regions known to disproportionately benefit from physical activity, are also linked to IL aptitude. Given that various types of IL rely on overlapping neural substrates to those affected by physical activity, we could predict a positive association between physical activity and IL in the TLT, in particular in initial stages of learning when the hippocampus may be most involved.

The prediction above, however, is complicated by findings suggesting that IL is impaired by the sustained engagement of the prefrontal cortex and hippocampus. For example, IL on a motor-sequence learning task improves following hypnosis [31], a procedure thought to disinhibit automatic behaviors by reducing the top-down modulatory influence of the prefrontal cortex on other brain regions. IL on a perceptual sequence learning task is negatively associated with dispositional mindfulness, a trait-like quality hypothesized to increase prefrontal and hippocampal functioning and to thus promote top-down regulatory processes [32]. In addition, better IL is associated with *decreasing* hippocampal and *increasing* striatal activation over the course of both motor and perceptual IL tasks, suggesting that optimal hippocampal involvement in IL is comparatively short-lived to that of the striatum during the acquisition phase of IL, even across tasks of differing motor demands [23,26]. This later finding suggests that optimal IL performance may be related to an earlier switch from hippocampal to striatal control during IL acquisition. That is, there is evidence that the hippocampal and striatal systems may compete for control of behavior during IL tasks, particularly initially in learning when the systems are observed to be most coactive (e.g., [23,26,28]).

Further, there is evidence from addiction studies in both animals and humans demonstrating that exercise reduces habitual (i.e., drug-seeking) behaviors, lowers the risk of addictive relapse, and even leads to a reduction in individuals’ propensity to develop new habitual behaviors (for review see [33]). Since better IL has been posited to be an indicator of inter-individual variability in one’s propensity for habit formation [34,35], it is possible that IL may not always be advantageous and that physical activity may be linked to worse IL due to its enhancement of neural systems supporting more deliberate, and explicit, learning strategies. Together, these findings suggest that physical activity may be *negatively* related to IL performance.

An additional open question stems from the fact that, to date, the majority of studies examining the link between physical activity and cognitive functioning have focused on older adults. It is therefore unclear to what extent PA relates to cognitive functioning—implicit or explicit—in other adult age groups, especially younger adults. Indeed, there are known moderators of the physical activity and cognition relationship (e.g., age, gender, dosage), such that the effects of physical activity on cognition are stronger for certain subgroups (e.g., older adults) than for others (e.g. [7]). Young adults are a generally healthy/high-functioning group that might thus be hypothesized to be less sensitive to the effects of physical activity. However, young adults, on average, have a remarkably sedentary lifestyle in contemporary society [36,37]. Thus, even though they may be in their cognitive “prime”, there is a wide range of inter-individual variability in physical activity engagement within this age group that might have important implications for understanding individual differences in cognitive performance and overall health. Therefore, a secondary aim of the present study was to test for relationships between physical activity and cognitive functioning in an adult sample much younger than that typically examined.

In the present study, fifty young adults underwent objective physical activity monitoring. They then completed a measure of IL, the Triplets Learning Task, as well as measures of relational memory and executive cognitive functioning (an explicit relational memory task and a word-color Stroop task). In regards to our primary aim, we predicted that IL in the TLT would be related to physical activity levels, with the directionality of this association possibly dependent on the session of learning. In regards to our secondary aim, we predicted that physical activity would be positively associated with relational memory and executive functioning, replicating the findings from older adults in a younger adult age group.

## Materials and Methods

### Subjects

Fifty healthy young adults (*M*±*SD* = 23.88±4.60 years old; 27 female) were recruited from the University of Pittsburgh and surrounding community to participate in a study assessing cognition and physical activity. Informed written consent was obtained in accordance with protocol and procedures approved by the University of Pittsburgh Institutional Review Board and the principles expressed in the Declaration of Helsinki.

### Procedure

Participants completed testing on three days, scheduled approximately one week apart. On the first and third days participants completed a battery of cognitive tasks and questionnaires; during the second day they completed a maximal fitness test on a treadmill and were fitted with a physical activity monitoring device equipped with an accelerometer to objectively measure physical activity. The device was worn for the intervening week between the second and third days. We focus on the accelerometry data in the present study. A subset of participants (N = 41) also completed a fourth, magnetic resonance imaging (MRI) session (results not reported here) in which an additional cognitive task (described below) was administered.

### Objective Physical Activity Assessment

#### Physical activity monitoring

To obtain objective measurements of physical activity (PA), all participants were fitted with a SenseWear^®^ armband and wore the devices for approximately one week (*M*±*SD* = 5.98±1.04 days). All participants wore the device on the back of the upper left arm (triceps region) and were instructed to wear it at all times, except when in presence of water (e.g., when showering, bathing or swimming). The devices were worn on average 88.31 ±11.15% of the total time, suggesting good overall compliance with these instructions. There were several main outcome measures from this assessment: (1) *average steps* per day, (2) hours of PA per day which is any activity of 3.0 Metabolic Equivalents (METs) or greater, hereafter referred to as *hours of PA*, and (3) number of PA bouts (of 3.0 METs and greater) lasting 10 continuous minutes or longer per day, hereafter referred to as *number of bouts*. To reduce the total number of statistical tests and because of high correlation between these measures, these three outcome variables were combined into a single composite PA measure (hereafter referred to as PA*) by averaging normalized values of each.

### Cognitive Testing

#### Implicit learning

**Fig 1:**
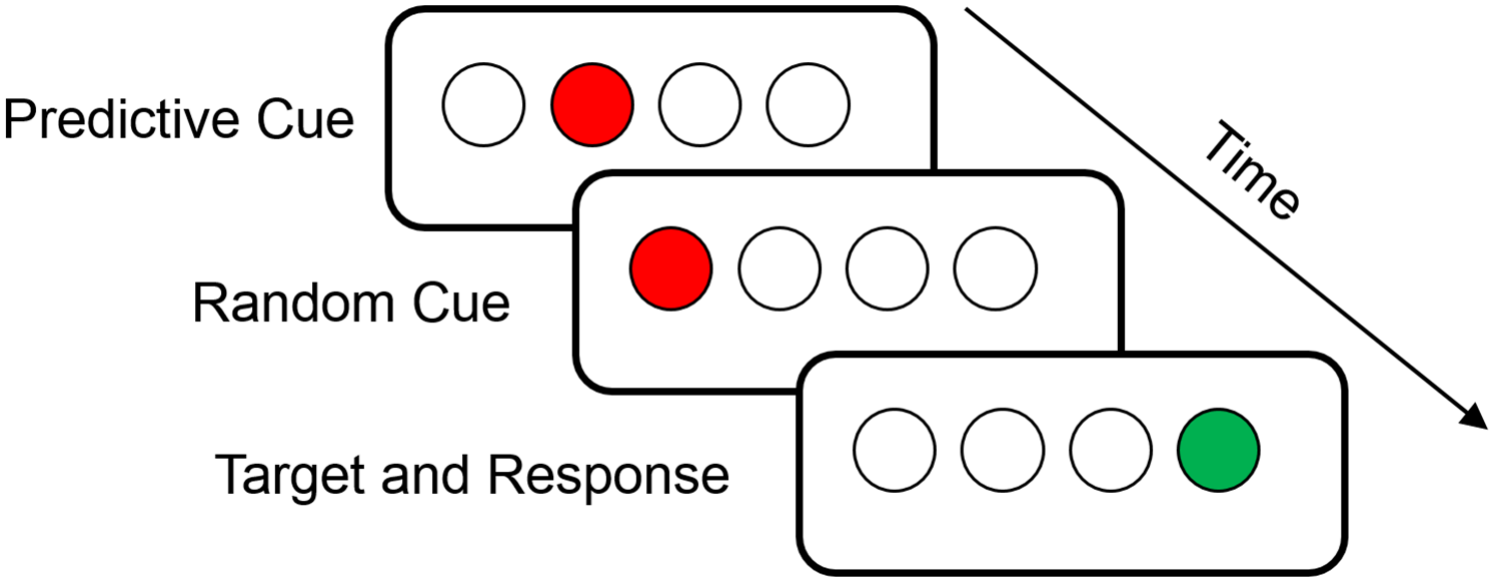
Schematic of the Triplets Learning Task. Each trial, or ‘triplet’, is comprised of two sequentially presented red cues and a green target. Participants observe the red cues and respond as quickly as possible to indicate the location of the green target. Unbeknownst to them, there is a probabilistic regularity embedded in the task, such that the first red cue predicts the target’s location with 80% probability.

To assess implicit learning, participants completed the Triplets Learning Task (TLT) [20]. A schematic of the TLT is shown in Fig 1. Participants view a horizontal row of four open circles centered on a computer screen. On each trial, a three-event sequence of the circles (a “triplet”) fills in sequentially red, red, and then green. Participants are instructed to observe the first two red “cues” and to indicate the location of the green “target” by pressing a spatially corresponding button. Importantly, participants are not informed of the presence of regularities in the task; they are only encouraged to respond as quickly as possible to the targets. Cues are displayed one after the other for 120ms each (150ms ISI) and are followed by the target 150ms later, which remains in view until participants make a correct response. The next trial begins 650ms after participants respond to the target.

Unbeknownst to participants, the TLT contains a probabilistic regularity such that the location of the first red cue probabilistically predicts the target’s location, and the location of the second red cue is random. In the present study, the target appeared in a single high probability (HP) location following the predictive cue on 80% of trials, while on the other 20% of trials the same red cue was followed by a target occurring in one of the three low probability (LP) locations. Because there are 64 triplets possible, this resulted in 16 triplets occurring with HP (80% frequency; each occurring 15–40 times per 500 trials) and 48 triplets occurring with LP (20% frequency; each occurring 1–8 times per 500 trials). The high probability triplets were not determined randomly, but rather each participant was assigned to view triplet sequences that followed one of 6 possible patterns (i.e., 1-2-3-4; 2-3-4-1; 3-4-1-2, etc.), where the numbers correspond to the possible spatial positions of the predictive (first) cue and target. S1 Table contains a list of all possible triplets, as well as their probabilistic designations in each of the 6 possible patterns. For example, a participant receiving the regularity 1-2-3-4 would see the triplets: 1r2, 2r3, 3r4, and 4r1 (where r indicates the position of the second, non-predictive red cue) with high probability, and all other triplets, such as 2r1, 3r2, 4r3, and 1r4 with low probability. This ensured that cue-target relationships were counterbalanced across participants and that all of the possible target locations occurred equally often, eliminating the possibility of target frequency-based learning.

On day 3, each participant completed two 500-trial sessions of the TLT (1000 trials total). The TLT is administered in sessions of 500 trials in order to prevent fatigue. In addition, in order to reduce the potential bias of individual differences in overall speed (e.g., from speed-accuracy tradeoffs), participants receive intermittent feedback every 50 trials in which they are told to either “focus more on speed”, “focus more on accuracy”, or “your responding is about right”. Unbeknownst to them, the feedback message is based on their accuracy on the proceeding 50 trials and is designed to guide all participants to respond with a similar error rate per session (~8%).

As in previous studies using the TLT, learning is quantified by calculating *Implicit Associative Learning (IAL) scores*, which measure learning unbiased by individual differences in overall reaction time [20,38–40]. Prior to calculating these scores, and consistent with the previous studies cited above, we eliminated certain types of trials from all analyses, including “repetitions” (e.g., 111, 333) and “trills” (e.g., 121, 343). We eliminated these because people have preexisting response tendencies to these types of trials and because (given the 6 possible patterns as described above) these triplets always occur with low probability across all participants. Thus, unlike all other triplets, their probabilistic designation is not counterbalanced across subjects [20,41,42]. We also eliminated inaccurate trials before calculating IAL scores.

To determine the IAL score for each participant, for each triplet, the median RT for all correct responses to that triplet is correlated with the number of times that triplet had actually occurred for that participant during each task session [20]. People who have stronger negative correlations between RT and triplet frequency are revealing more sequence-specific learning in that they are responding faster to triplets that occur with greater frequency (and thus have more predictable targets) (See S1 Fig for representative scatterplots of two participants). For ease of interpretation, the correlations were multiplied by -1 prior to inferential analyses so that higher IAL scores reflect greater learning. IAL scores were calculated for each participant, for each session of training (i.e., per every 500 trials of the TLT). This calculation was done based on prior work using the TLT, which has found that IL in early vs. later sessions of the TLT often show different patterns of effects [23,43]. One reason proposed for this is that early and later sessions involve slightly different neural substrates. In fact, there is functional magnetic resonance imaging (fMRI) evidence that the earliest learning session of the TLT may rely more on the medial temporal lobe than later learning, when the caudate nucleus is thought to dominate (e.g., [23]). Given this prior evidence, we considered it theoretically important to examine scores from the first and second sessions of the TLT separately (but see S2 Table for results collapsed across TLT sessions).

Of note is the fact that learning in the TLT can also be observed via accuracy. However, we chose to focus only on reaction time here. One reason for this is that response accuracy in the TLT is typically very high, making it more difficult to detect differences in accuracy across the HP and LP conditions. Another reason is that the occasional performance feedback messages given throughout the TLT is designed to drive participants to respond with similar error rates (~8%) in order to reduce the influence of individual differences in overall speed and/or speed-accuracy tradeoffs when comparing learning across individuals and groups. Because the range of accuracy is compressed by this feedback, accuracy-based scores are a less sensitive measure of learning compared to RT-based measures in the TLT.

Following the completion of the TLT, participants completed a computerized recognition task in which they were shown all 64 possible triplets that had occurred during training and were asked to rate how often they thought each occurred (1 = infrequently; 2 = frequently).

#### Relational memory

We used a test of explicit relational memory, employing the variant of the spatial reconstruction task [44] described in detail by Monti and colleagues [45]. The task involves relational memory binding, and several different variants of the task, including especially the outcome measure “swaps” or “swap errors” (see below), which have been shown to be highly sensitive to the structural integrity and/or volume of the hippocampus [44,45] as well as to the beneficial effects of physical fitness [46]. Briefly, on each trial of the task participants study the spatial arrangement of five novel line drawings and are told to remember the arrangement for a later test. Study time is self-paced, and participants are instructed to use the mouse to click on each stimulus, which initiates the start of the test phase. Following the study phase, there is a 4000ms delay in which participants see a blank screen; a self-paced test phase begins after this delay. In the test phase, stimuli appear aligned at the top of the screen, and participants use the mouse to click and drag them to where they were positioned during the study phase. Participants completed three practice trials and 15 experimental trials (2000ms ITI).

Memory errors committed during the test phase were the primary outcome measures from the spatial reconstruction task. Errors were assessed using 4 metrics: (1) average *item misplacement* (in pixels), (2) *edge resizing* (in pixels), (3) *edge displacement* (in radians), and (4) *swaps* (proportion of all possible pairwise relationships). Detailed descriptions and examples of the various errors are provided in Watson et al., [44]. In all cases, higher values indicate worse memory performance.

#### Executive functioning

To measure executive functioning, we used a color-word Stroop task. In the task, participants view a series of words presented in red, green, or blue font and are instructed to respond as quickly as possible to indicate the color of the word, while ignoring the word’s semantic identity. Participants made their responses using a scanner-compatible response glove. They indicated the color of the words by pressing the index, middle, and ring finger of their dominant (right) hand, which represented the colors, red, blue, and green, respectively. There were three stimulus conditions: (1) Incongruent, in which the word names a different color than the ink color in which it is printed (e.g., the word ‘‘red” in blue ink); (2) Congruent, in which the word matches the ink color in which it is printed (e.g., the word ‘‘red” printed in red ink); and (3) Neutral, in which the word is unrelated to color (e.g., the word ‘‘book” in red ink).

In the present study, the Stroop task was administered during and MRI scan. Stimuli from each condition were presented in an event-related manner, such that trials from the three conditions were intermixed. This presentation format with intermixed trials has been shown to minimize the use of strategies and/or development of attentional task sets (e.g., [47]). Participants completed several practice trials of the task outside the scanner and 10 additional practice trials inside the scanner (i.e., prior to the experimental block) to ensure they were comfortable with the response glove and understood the response mappings. Participants then completed 120 trials (40 per condition) comprising the experimental block inside the scanner.

The primary outcome from the Stroop test was the magnitude (in ms) of the *Stroop effect*, a measure of the degree of interference between an automatic process (word reading) and the current task goal (color naming). To calculate the Stroop effect, each participant’s average reaction time on congruent trials was subtracted from that on incongruent trials, and then divided by the RT on congruent trials [48]. Larger values indicate a higher percent interference from the conflicting color-word information in incongruent trials compared to the consistent color-word information in the congruent trials. Higher values of the Stroop effect therefore indicate poorer executive functioning.

### Statistical Approach

All variables were first examined for normality of distribution; the Stroop effect, as well as the item misplacement and swap error measures (from the relational memory task) were significantly positively skewed and were thus log transformed prior to analysis. Associations between PA* and cognitive performance were then assessed using linear regression. Age and gender were entered as covariates in the regression models because of their association with physical activity and several of the cognitive measures. In addition, given evidence suggesting that gender may be an important moderator of the effects of exercise on cognitive functioning (e.g., [7,49]), we also included a PA* x gender interaction term in the models. We followed up on any significant PA* x gender interactions by conducting gender-stratified regressions (controlling for age).

In secondary analyses, we examined the relationship between each individual PA measure comprising the PA* composite and cognitive performance. All analyses were conducted using IBM SPSS 23. Statistical significance was estimated using bootstrapping methods with 5,000 resamples and thresholded at p <.05.

## Results

### Implicit Learning

#### TLT performance

One participant was eliminated from the TLT analyses because of response accuracy below chance. Accuracy for the remaining sample (N = 49) was high (*M*±*SD* = .93±.03), so few trials were eliminated prior to the calculation of IAL scores. One sample t-tests revealed that IAL scores for both the first (*M*±*SD* = .16±.08) and second (*M*±*SD* = .20±.13) session of training were significantly above zero, suggesting that, on average, participants successfully learned the regularity (*t*s>10.98, *p*s < .001). Scores from the second session were significantly higher than those from the first session of training demonstrating that learning increased over the course of the task, *t*(48) = 2.27, *p* = .03.

#### TLT explicit recognition performance

In order to examine whether learning on the TLT was implicit, participants’ mean ratings on the computer-based recognition task were entered into a one-way ANOVA with triplet type (HP, LP, reps, and trills) as a repeated measures factor. There was no effect of triplet type, *F*(3, 138) = .647, η_p_^2^ = .03, *p* = .56, demonstrating that ratings did not differ across the various types of triplets presented during the task. Participants were therefore unable to reliably make the critical distinction (i.e., between HP and LP triplets) when explicitly asked to do so, supporting that learning in the TLT was implicit.

#### PA* and implicit learning

**Fig 2:**
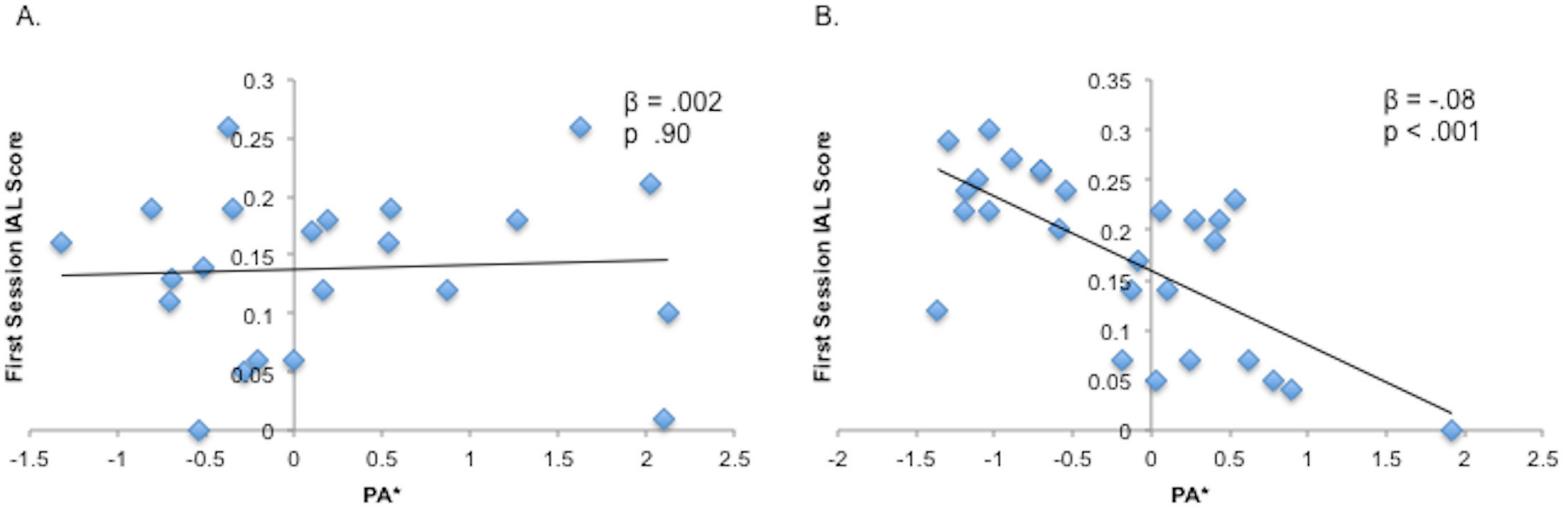
Gender moderates the relation between PA and implicit learning. Plots depict correlations between IAL scores from the first session of training and a composite measure of PA in males (A) and females (B).

All effects of PA* on IL were specific to TLT scores from the first session of training; there were no significant effects for any of the planned analyses on TLT scores from the second session of training. Therefore, we use “IL” to refer to TLT scores from the first session in the following sections but report associations with scores from both sessions of the TLT in Table 1. After accounting for the variance explained by age and gender, there was no effect of PA* on IL performance (*β* = -.08, *p* = .95). However, the relationship between PA* and IL was significantly moderated by gender (PA x gender: *β* = .08, *p* = .004). Given this significant interaction, we stratified the sample by gender in order to probe its nature. As shown in Fig 2 and Table 2, there was a significant negative effect of PA* on IL in females (N = 27; *β* = -.08, *p* < .001), but not in males (N = 22; *β* = -.002, *p* = .92).

**Table 1:** Relationship between PA*, the three measures comprising PA* (average steps, hours of PA, and number of bouts), and implicit learning performance.

	Implicit Learning	
	First Session	Second Session
PA*	.001	-.026
PA* x Gender	-.08*	-.022
Avg Steps	-5.7E-7	2.8E-6
Avg Steps x Gender	**-1.4E-5**	-6.6E-6
Hrs PA	.002	-.022
Hrs PA x Gender	-.05*	-.004
Bouts	.002	-.011
Bouts x Gender	-.03*	-.009

Beta values reported.

Bolded values marked with an asterisk denote significant effects at p < .05, and bolded values without an asterisk denote marginal effects.

Age and gender were included as covariates in all models.

**Table 2:** Relationship between PA*, the three measures comprising PA* (average steps, hours of PA, and number of bouts), and explicit cognitive performance.

	Explicit Relational Memory				Executive Control
	item misplace	edge resize	edge distortion	swaps	Stroop effect
PA*	-13.29	-.06	-.01	-.26*	-.39*
PA* x Gender	**__**	**__**	**__**	**__**	**__**
Avg Steps	-.01	2.9E-5	-5.0E-6	-8.6E-5*	-7.8E-5*
Avg Steps x Gender	**__**	**__**	__	**__**	**__**
Hrs PA	-4.64	-.014	-.004	-.12	-.25*
Hrs PA x Gender	__	__	__	__	**__**
Bouts	-1.84	-.01	-.002	-.07	-.13*
Bouts x Gender	__	__	__	__	**__**

Bolded values marked with an asterisk denote significant effects at p < .05, and bolded values without an asterisk denote marginal effects.

Age and gender were included as covariates in all models.

Beta values are not provided for models in which the interaction term was dropped.

To examine the possibility that males and females learned at different rates (rendering the IAL scores by session less reliable in males than females), we recalculated the IAL scores for every 200 trials to create 5 “Epochs”. A graph of IAL scores by Epoch split by gender is shown in S2 Fig. While the division of the data into 200-trial increments is arbitrary, we reasoned that scoring the data in this way would allow us to more accurately plot the shape of learning and obtain an estimate of learning rate for each gender separately. We compared learning rates across the epochs using a mixed effects repeated measures ANOVA with gender as the between-subjects factor and Epoch as the repeated factor. There was no gender x Epoch interaction, *F*(1,46) = 1.03, *p* = .32, suggesting that learning rates did not differ between males and females.

We also examined whether males and females differed in PA levels in general, as this could potentially inflate the relationship between PA and cognition in the more active group. There were marginal differences between males and females in PA* (*t*(47) = 1.83, *p* = .07), Average Steps per day (*t*(47) = 1.72, *p* = .09), and Bouts per day (*t*(47) = 1.78, *p* = .08). In all cases, males were marginally more active than females. There were no gender differences in Hours of PA per day (*t*(47) = 1.51, *p* = .14). Given this pattern it seems unlikely the relationships we report within the gender groups are due to group differences in average PA levels.

Given the significant interaction described above with the composite PA measure, we conducted secondary exploratory analyses to examine whether the associations described above were being driven equally by all PA variables or were bring driven by a subset of the PA variables (Table 1). There was no main effect of any of the PA variables on IL controlling for age. However, there was once again evidence that gender moderated the relationship between PA and IL; the *average hours* x gender (*β* = -.05, *p* = .002), and the *number of bouts* x gender (*β* = -.03, *p* = .001) terms were significant predictors of IL, and the *average steps* x gender term was a marginal predictor (*β* = -1.3 x 10^−5^, *p* = .08). In all cases, the nature of these interactions was such that there was a negative effect of PA on IL in females, such that a greater amount of PA was associated with less IL, but no effect in males (Table 3).

**Table 3:** Gender-specific relationships between PA*, each of the three PA measures (average steps, hours of PA, and number of bouts), and IL. Bolded values marked with an asterisk denote significant correlations, and bolded values without an asterisk denote marginal correlations.

**Females**		
	First Session	Second Session
PA*	-.08*	-.04
Avg Steps	-1.4E-5*	-5.6E-6
Hrs PA	-.05*	-.02
Bouts	-.03*	-.02
**Males**		
PA*	.002	-.03
Avg Steps	-6.1E-7	-3.1E-6
Hrs PA	.002	-.03
Bouts	.002	-.01

Males had marginally higher PA*, Avg. Steps, and Bouts than females. There were no gender differences in Hrs. PA. Age was included as covariate in all models.

### Relational Memory

#### Spatial reconstruction task performance

Data from two additional participants was lost due to experimenter error, thus the final sample size for these analyses was N = 47. Participants committed all four types of errors significantly more than would be expected due to chance (one sample t-tests: all *t*s> 9.51, *p*s < .001), thus we examined all of them in relation to PA.

#### PA and relational memory

There was no evidence that gender moderated relational memory performance; the PA* x gender interaction term was not a significant predictor of any memory performance measures (i.e., misplacement, log transformed edge resizing, distortion, or log transformed swap errors). The interaction term was thus dropped from subsequent analyses. After accounting for the variance explained by age and gender, there was a significant negative effect of PA* on (log transformed) swap errors (*β* = -.26, *p* = .04). Since there were no effects of PA* on any of the other relational memory measures (*p*s > .53), we focus on swap errors for the secondary analyses reported below.

We examined the contribution of each individual PA variable to swap errors. There was a significant negative effect of *average steps* (*β* = -8x10^-5^, *p* = .04), such that a higher number of average steps per day predicted fewer swap errors. There was no effect of *number of bouts* (*β* = -.07, *p* = .14) or *hours of PA* on swap errors (*β* = -.12, *p* = .16). Thus, the main effect of PA* on swap errors reported above appears to have been driven largely by the average steps measure.

### Executive Functioning

#### Stroop performance

The Stroop task was administered during the MRI session, and so the analyses we report include only the subset of participants (N = 41) who completed the MRI. Participants were highly accurate on the task (*M*±*SD* = .97 ± .04), thus the Stroop effect was calculated using participants’ reaction times. A paired sample t-test showed that reaction times to incongruent trials were significantly slower (*M*±*SD* = 730.49±75.32) than those to congruent trials (*M*±*SD* = 645.57 ± 63.94; *t*(40) = 10.73, *p* < .001), suggesting that there was a Stroop effect in the sample on average. We next examined whether individual differences in the magnitude of the Stroop interference effect was correlated with PA.

#### PA* and stroop performance

As with the relational memory analyses, there was no evidence that gender moderated Stroop performance; the PA* x gender interaction term was not a significant predictor of the (log transformed) Stroop effect (β = 0.05, p = .90) and was thus dropped from subsequent analyses. After accounting for the variance explained by age and gender, there was a significant negative effect of PA* on the Stroop effect, such that higher physical activity was associated with lower Stroop interference (*β* = -.39, *p* = .02). Next, we tested the contribution of each individual PA variable to this effect. There were significant negative effects of all three PA variables: *average steps* (*β* = -7.8 x 10^−5^, *p* = .04), *hours of PA* (*β* = -0.25, *p* = .03), and *number of bouts* (*β* = -.13, *p* = .03) on the Stroop effect (Table 2).

### Additional Exploratory Analyses

The negative relationships between PA and IL reported above are consistent with the idea that exercise may decrease one’s propensity for forming habits through implicit learning. If this is the case, then individual differences in IL might be related to certain health outcomes (e.g., body mass index; BMI) that are manifestations of habitual behaviors in domains of everyday life (e.g., eating habits). To explore this possibility, we conducted exploratory regressions examining the relationship between BMI (calculated from objective height and weight data collected during the fitness testing session with Health O Meter 500KL Digital Scale), and (1) PA* and (2) IL, controlling for age and gender in each.

There was no evidence that gender moderated the relationship between BMI and PA*; the BMI x gender interaction term was not significant (*β* = -0.01, *p* = .82) and was consequently removed from analyses. As expected, however, there was a negative relationship between BMI and PA*, such that lower BMI was associated with higher PA (*β* = -0.06, *p* = .03).

We next examined the relationship between BMI and IL. There was also no evidence that gender moderated the relationship between BMI and IL; the BMI x gender term was not significant (*β* = .006, *p* = .01) and was again removed from analyses. However, there was a significant positive effect of BMI on IL, (*β* = .006, *p* = .01), suggesting that those with a higher BMI (perhaps an indication of poorer eating habits) also tended to have greater propensity for IL.

## Discussion

The primary aim of the present study was to examine how PA relates to an implicit cognitive function, IL. Further, we examined whether previously reported relationships between PA and explicit cognitive functioning could be replicated in an adult sample much younger than typically tested. Our results demonstrate for the first time a dissociation in the relationship between PA and explicit cognitive functioning vs. IL. Specifically, PA was associated with *better* performance on more explicit cognitive tasks for both genders, but *worse* performance on an implicit probabilistic sequence learning task for females only.

### PA and Explicit/Executive Cognitive Functioning

The correlations we observed between PA and performance on the explicit relational memory and executive cognitive tasks are in directions consistent with those previously reported in the literature [4,5,46,50]. That is, as we predicted, more PA was associated with fewer errors on the relational memory task, as well as with less interference on the Stroop task. The fact that these relationships are consistent with earlier research is important as it helps to rule out other potential explanations for the negative correlation we observed between PA and IL, such as the possibility that unique characteristics of our sample (and not IL per se) were responsible for the effect.

Importantly, however, the present study goes beyond the existing research by demonstrating that relationships between PA and explicit/executive cognitive functioning—the cognitive domains showing the largest improvements in training studies of PA in older adults [7]—extend to a younger adult sample with no cognitive impairments. Thus, cognitive performance is sensitive to individual differences in PA, not only in populations with known cognitive deficits, but also to those in their cognitive prime.

### PA and Implicit Learning

The primary and most novel result from the present study was that PA was negatively associated with IL, but only in females. The finding that gender moderated the effect of PA on IL is consistent with previous PA research, which indicates that PA may have gender-specific effects on cognitive functioning. Sexually dimorphic effects of PA on cognitive functioning have most often been shown to favor females over males. For example, Baker and colleagues [50] found that, despite comparable improvements in cardiorespiratory fitness following a 6-month aerobic exercise intervention, only females showed improved executive functioning (using a Stroop task similar to that used here). Aerobic exercise had no effect on executive functioning in men. In addition, a large prospective study of middle aged adults (The Canadian Study of Health and Aging) found that greater baseline PA was associated with a lower risk of future cognitive impairment in females, but not in males [51]. Both of these findings are consistent with an earlier meta-analysis of aerobic exercise interventions in older adults, which reported that studies comprised of proportionately more women tend to show larger exercise-related cognitive improvements compared to those comprised of more men [7]. The negative PA-IL relationship we observed in females is therefore in line with the gender differences previously reported in the PA literature (but see [52] for a different effect in children). Given that we did not observe similar gender-specific effects for the explicit functions, these results may also indicate that the sexually dimorphic effects of PA are especially prevalent for IL. Although only speculation at this point, one possible explanation for this is that striatal-dependent cognitive processes are more sensitive to gender effects than those dominated by other neural systems. More studies using a variety of cognitive paradigms are necessary, however, to further investigate this possibility.

One potential alternative explanation for the PA x gender interaction we observed is that male participants simply learned at a different rate than females; thus the median split could have rendered the association between PA and implicit memory more unreliable in males compared to females. We examined this possibility post-hoc by breaking down the IAL score calculation into smaller increments and comparing the trajectory of learning in males and females (S2 Fig). We found no evidence that learning rates differed based on gender, and so differences in learning rate cannot explain the pattern of results we report.

It is important to acknowledge that several recent studies *have* examined the effects of acute [53–55] or long-term [56] exercise on motor learning, including motor sequence learning, and these studies have reported findings that differ from those reported here. For example, some of these studies have found that moderate-high intensity acute exercise or longer-term exercise training leads to improvements in implicit motor learning [54–56], while one found no effects [53]. However, the present results may not be as inconsistent with these earlier studies as they seem, because the handful of prior studies involving PA focused on *motor*-based implicit learning; perceptual IL has not been examined in relation to PA. In contrast, the task we employed, the TLT, does not contain any motor sequence, in that people respond only to the target (and not to the predictive cue) on each trial. Importantly, such perceptual sequence learning, differs from motor-sequence learning because it involves extracting subtle statistical regularities from the environment without the confounding influence of motor fluency/capacity [21]. Crucially, more perceptual forms of sequence learning may have slightly different neural underpinnings compared to motor-based sequence learning (e.g., motor-based sequence learning may rely more on the hippocampus than perceptual sequence learning) [57]. Further, different subregions of the striatum are known to support more motor (e.g., putamen) vs. more cognitive (caudate) processes [58]. It will therefore be important for future studies to directly compare the effects of PA on perceptual vs. motor-based versions of probabilistic sequence learning tasks to determine when PA helps versus hinders different variations of implicit learning processes. Such distinctions could have important implications in rehabilitative contexts.

### Neural Underpinnings

Taken together, the present pattern of results is consistent with findings from neuroimaging studies of PA. These studies suggest that PA exerts its salutary effects on physical and cognitive health by increasing structure (e.g., regional brain volume) and promoting more efficient functioning (e.g., activation) of medial temporal and frontal brain regions supporting explicit and executive cognitive functions [4,14,15]. The positive correlations between PA, explicit memory, and executive functioning we observed support this mechanistic hypothesis (see [59] for a review).

The PA-IL results are also consistent with a neural mechanism involving increased hippocampal and prefrontal functioning and, further, indicate that there may be tradeoffs to the disproportionately enhanced functioning of these neural regions and the cognitive functions they support (e.g., more explicit performance strategies). Specifically, the present results join recent findings from the implicit learning literature using the TLT or similar implicit probabilistic sequence learning tasks, which demonstrate that enhanced functioning of hippocampal and frontal brain regions may not be beneficial to IL [60,61,31]. Indeed, there is evidence suggesting that there can be an antagonist relationship between cognitive functions supported by the hippocampus and frontal cortex and those supported (or at least optimally dominated) by the caudate nucleus [23,26]. In other words, there might be competition between two determinants of behavioral performance and the neural systems that support them [25]. In the case of tasks such as the TLT, for example, explicit strategies supported by the hippocampal system may dominate initially in learning as rapid associations are being formed but, due to the probabilistic nature of the cue-target relationships, superior learning is observed when the striatal system dominates [23]. Consistent with this idea, an experimental manipulation that *decreases* functional connectivity of frontal brain regions to the rest of the brain (e.g., hypnosis) *increase* IL performance [31]. Thus, there is evidence for competition between neural systems at during IL.

In addition, in the case of implicit acquisition of new regularities (the type most relevant to the present study), a pattern of decreasing hippocampal and gradually increasing caudate activation over the course of IL (including in the TLT) is typically associated with better implicit perceptual, as well as motor, learning in younger adults [23,26]. Thus, one interpretation of the present results, albeit only speculative at this point, is that people who are less physically active have lower functioning of hippocampal (and perhaps also prefrontal) regions compared to people who are more physically active. While lower levels of PA may have led to poorer performance on explicit/executive cognitive tasks, it may have enabled the task-optimal striatal system to dominate more quickly during the TLT, thus leading to superior IL. The fact that the negative PA-IL relationship was specific to the first session of IL, a period when the hippocampus is most active in IL and may compete with the caudate for control of behavior [23,26,28], supports this idea.

In line with this interpretation, research using animal models demonstrates that PA modulates the relative involvement of memory systems (e.g., by boosting the relative involvement of the hippocampal system), and that estrogen levels further influence PA-memory system interactions, such that higher estrogen might promote more hippocampal system involvement [62–64]. Thus, findings from animal studies further support our hypothesis of why the gender-specific associations we observed between PA and implicit learning were limited to the first session of training in the TLT. Future work in humans could examine the pattern of regional brain activation during an IL task in sedentary compared to a more physically active groups of males and females to further explore these interpretations.

The above interpretations regarding the moderating role of gender are mostly speculation because the precise mechanisms by which estrogen may moderate the effects of PA on cognition in humans are still unclear. However, gender differences in the effects of PA on cognition have been reported in the literature, including in recent work from our lab [65]. Studies have speculated that estrogen regulates the physiological and cardiovascular effects of PA making females more sensitive to the effects of PA compared to males [7,64,66,67]. Interestingly, estrogen might also influence PA levels [66], suggesting that the mechanisms for gender differences may be bidirectional and complex.

### Possible Health Implications

Given that a physically active lifestyle is generally considered to be beneficial for physical and more explicit cognitive functioning, it seems counterintuitive that it would be associated with impaired IL. After all, this type of learning is thought to underlie essential everyday functions, such as the development of new procedural skills and habits [19]. However, not all habits are adaptive. It is therefore possible that a negative relationship between PA and IL could benefit health in certain contexts or in certain populations.

In the case of addiction, for example, automatized and compulsive drug seeking behavior is thought to reflect hyperactivation in neural circuits supporting habit formation (e.g., striatal reward circuits) and hypoactivation in those supporting cognitive control (e.g., prefrontal and hippocampal regions) [33,68]. The prefrontal cortex and hippocampus—regions known to be underactive in addiction—are disproportionately upregulated by PA [14]. This suggests that PA may lead to a reduction in unhealthy behaviors, such as addiction, that may be associated with IL. Indeed, there is evidence that PA may be an effective treatment for addiction in that it reduces responses based on habitual (drug-seeking) behaviors, thereby lowering the risk of addictive relapse, and leading to a reduction in individuals’ propensity to form new dependencies [33]. In addition, mirroring the sex differences reported in the effects of PA on cognition discussed above, PA interventions for addiction are often more effective in females than in males, suggesting similar mechanistic underpinnings [49,69]. Thus, one possible health implication for the negative PA-IL relationship we observed in our study is that increased levels of PA lead to a reduced propensity for this type of learning (particularly in females), and that this might lead to less habitual, more conscious behavior in real-life contexts. Here we refer to “habits” as habitual behaviors that have become an automatic or compulsory response to particular, regularly encountered, contexts. While the motor patterns of a frequently practiced behavior (e.g., running) could indeed proceed in an automatic fashion (and thus be considered habitual themselves), the choice to exercise is usually explicit and effortful. Of course, the interpretation outlined above is difficult to assess with the present data but will be important to test in future studies, as it could be the case that not all habitual behaviors are created equal in terms of their implications for promoting health.

Additional support for the potential health benefit of the negative PA-IL relationship comes from our exploratory analyses on the relationships between PA, IL, and BMI. There was a positive correlation between IL and BMI and a negative association between PA and BMI in our sample. That is, those who were less physically active tended to be better implicit learners and to also have a higher BMI. This pattern of associations raises the possibility that one’s propensity for IL may be a marker for the tendency to form and rely on habits in everyday life (i.e., while eating). We did not collect any direct measures assessing habitual behaviors in the present sample. However, an interesting direction for future work would be to assess how basic cognitive processes, such as implicit learning, relate to more complex real-world behaviors, such as habitual eating patterns or substance abuse/dependence, and whether individuals’ propensity for these behaviors relates to their physical activity levels.

### Limitations

There are several limitations to the present study. First, the relationships we report are correlational, so it is not possible to infer whether engaging in more PA *caused* participants to learn more slowly on the TLT, or whether there is a confounding factor driving the novel negative relationship we observed. For example, a previous study using a task similar to the TLT demonstrated that those with more years of experience as musicians and video-gamers tended to attain higher IL scores, suggesting that expertise in certain musical and other domains could modulate one’s sensitivity to probabilistic regularities [70]. Given that we did not screen for musical or video game expertise in the present study, this could potentially confound the present findings. However, as is the case here, this earlier study was correlational in nature, and so it is also possible that people who are inherently good implicit learners are more likely to gain musical or video-game expertise, or that some third factor drives the relationship. In addition, it is important to note, that the causal link between PA and cognitive functioning *has* been established in the explicit domain—i.e., explicit functions improve following randomized controlled trials of PA [4,5,46,71]. Nonetheless, training studies in which sedentary participants are tested on an IL task both before and after PA training are necessary in the implicit domain as well, in order to clarify the issue of causation in the implicit domain as well.

Second, while we found no evidence of explicit awareness here, previous studies of the TLT have included a verbal interview in addition to the computer-based recognition task [20,38,43,72]. Including additional measures of explicit awareness in future studies would help rule out the potential alternative interpretation that some individuals (or groups) rely on explicit strategies more than others. However, unlike in prior sequence learning tasks, the probabilistic regularity in the TLT is very subtle and difficult to detect. In fact, explicit awareness has not been detected in this task, even in studies using multiple measures to assess awareness [20,38,43,72]. Further, giving participants explicit knowledge of the regularity in the ASRT, a task that is highly similar to the TLT in terms of its probabilistic structure, does not enhance their performance [61]. Thus, it seems unlikely that differences in strategy use or explicit knowledge could explain the pattern of results we report.

Finally, another limitation of the present study is that it investigated the relationship between PA and one type of implicit learning (probabilistic perceptual sequence learning). It is therefore not possible to determine whether PA would also be negatively associated with other forms of implicit learning (e.g., implicit context learning). It would therefore be informative for future studies to examine the specificity of the negative PA-IL relationship across different types of implicit learning tasks. Doing so could further our understanding of basic cognitive mechanisms underlying real-life habitual behaviors, such as addiction, that are known to decrease following PA.

### Conclusions

Despite these limitations, the present results raise the intriguing possibility that PA exerts its salutary effects on health by upregulating some cognitive processes, while downregulating others. Our results also highlight the importance of examining potential moderators of the effects of PA on cognitive functioning. From a public health perspective, identifying such factors is crucial for developing a better understanding of individuals most likely to respond to PA interventions and to develop more personalized treatment plans for maximizing health outcomes.

## Supporting Information

S1 Fig Relationship between median reaction time (RT) and triplet frequency for two representative participants. Participants are expected to get selectively faster to the High Probability (HP) compared to Low Probability (LP) triplets. More *negative* correlations between RT and triplet frequency therefore indicate more learning. IAL scores are the magnitude of this correlation multiplied by -1 (i.e., so that higher values reflect *more* learning).
(TIF)

S2 Fig IAL scores over 200-trial “Epochs” split by gender. Learning rate did not differ between males and females.
(TIF)

S1 Table List of all possible triplets TLT, as well as their High Probability (HP) and Low Probability (LP) distinctions in each of the 6 possible to-be-learned patterns. Reps and Trills are always LP and are highlighted in gray.
(DOCX)

S2 Table Relationship between PA*, the three measures comprising PA* (average steps, hours of PA, and number of bouts), and implicit learning performance collapsed across task sessions. Effects reported in the paper are specific to the first session.
(DOCX)
