# Supplementary figures and images for: Physical Activity Is Associated with Reduced Implicit Learning but Enhanced Relational Memory and Executive Functioning in Young Adults
Source: PLoS One. 2016 Sep 1;11(9):e0162100. doi: 10.1371/journal.pone.0162100 (PMC5008769; doi:10.1371/journal.pone.0162100)

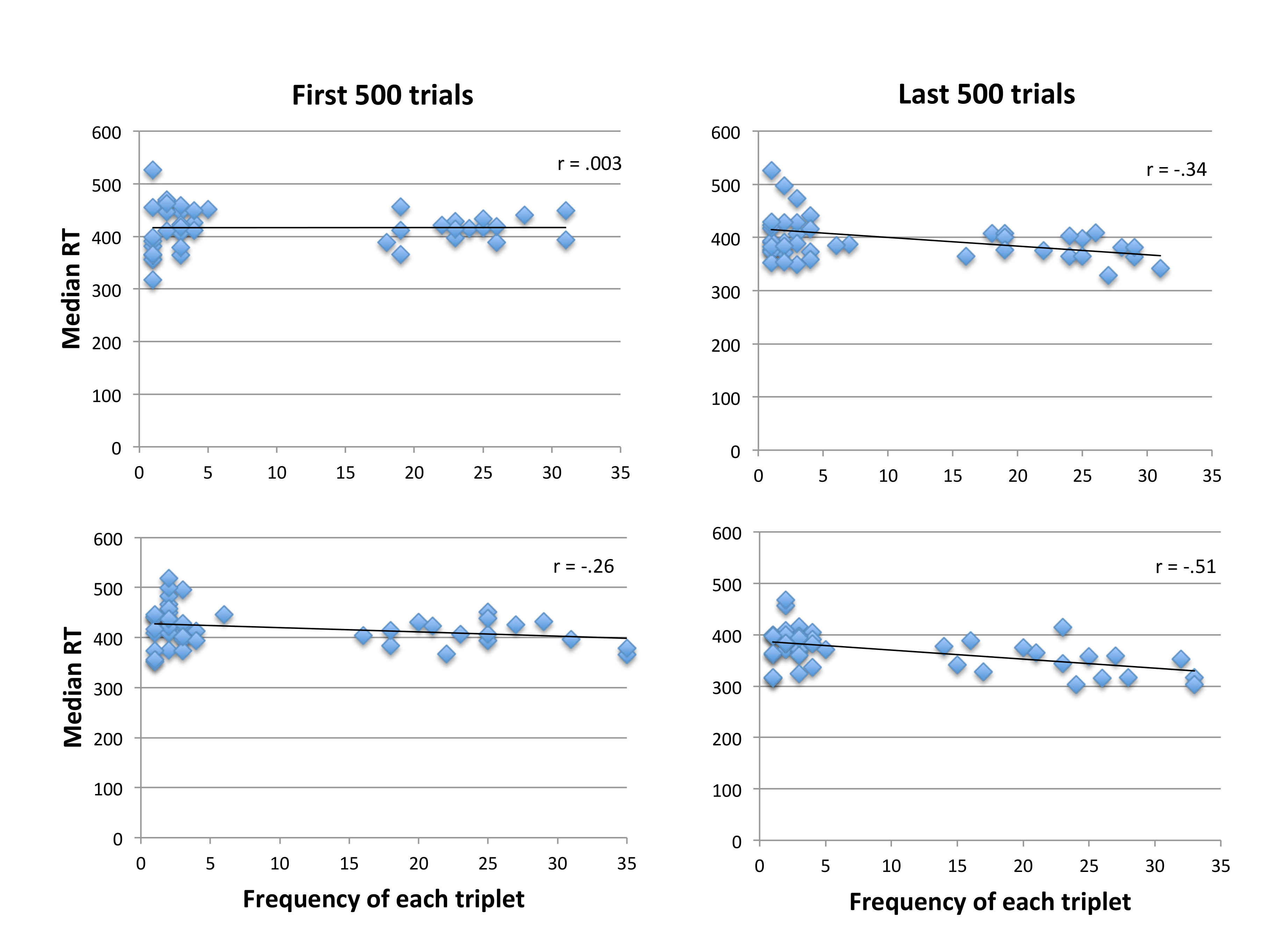

Supplement: S1 Fig — Participants are expected to get selectively faster to the High Probability (HP) compared to Low Probability (LP) triplets. More negative correlations between RT and triplet frequency therefore indicate more learning. IAL scores are the magnitude of this correlation multiplied by -1 (i.e., so that higher values reflect more learning). (TIF) [file pone.0162100.s001.tif]

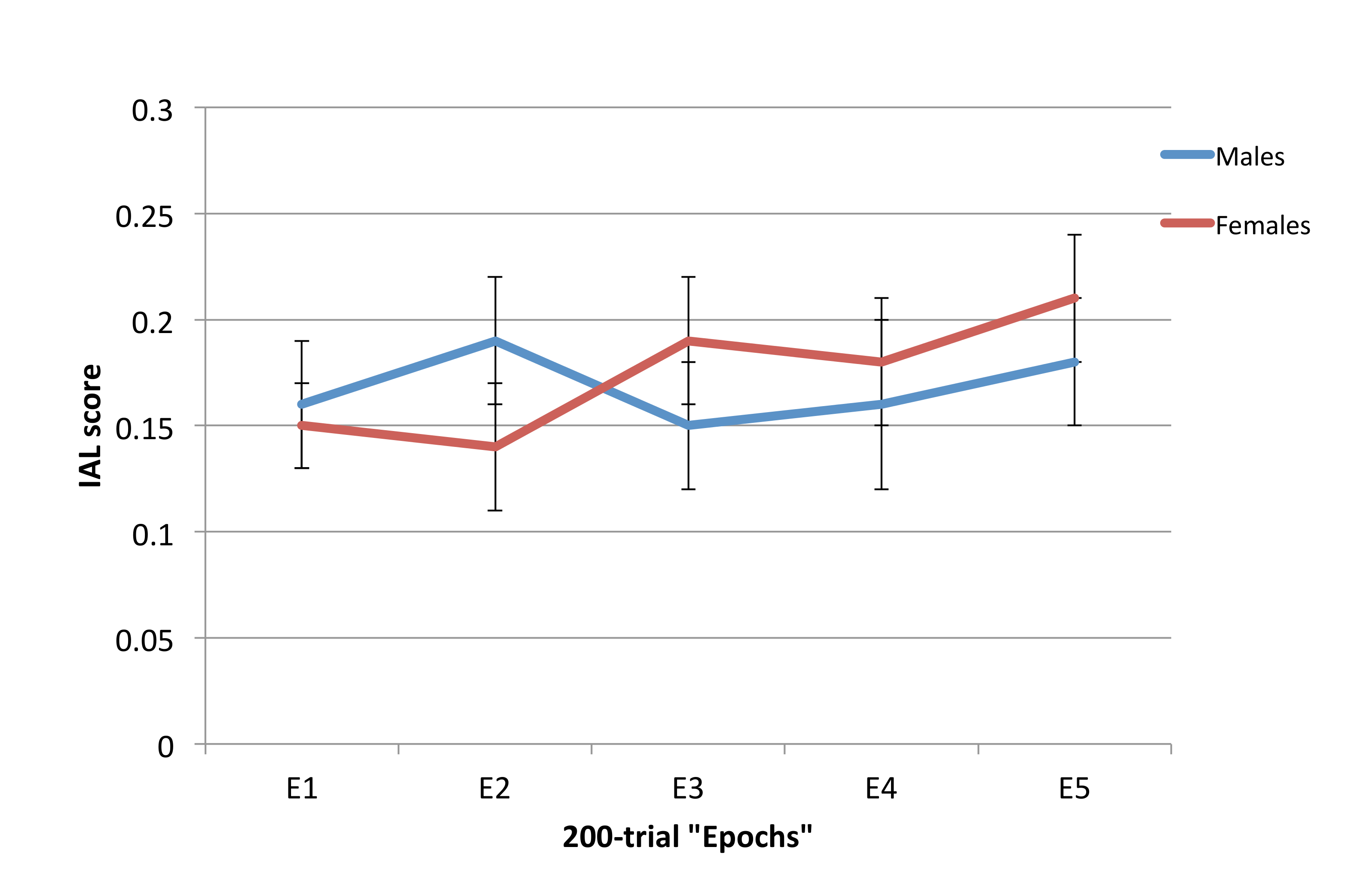

Supplement: S2 Fig — Learning rate did not differ between males and females. (TIF) [file pone.0162100.s002.tif]
