# Supplementary material for: Physical Activity Is Associated with Reduced Implicit Learning but Enhanced Relational Memory and Executive Functioning in Young Adults
Source: PLoS One. 2016 Sep 1;11(9):e0162100. doi: 10.1371/journal.pone.0162100 (PMC5008769; doi:10.1371/journal.pone.0162100)
Supplement: S1 Table — Reps and Trills are always LP and are highlighted in gray. (DOCX) [file pone.0162100.s003.docx]

**S1 Table.** List of all possible triplets TLT, as well as their High Probability (HP) and Low Probability (LP) distinctions in each of the 6 possible to-be-learned patterns. Reps and Trills are always LP and are highlighted in gray.

| **Triplet** | **Second Order** | | | | | | **Rep/Trill** |
| --- | --- | --- | --- | --- | --- | --- | --- |
|  | **1r2r3r4r** | **1r2r4r3r** | **1r3r2r4r** | **1r3r4r2r** | **1r4r2r3r** | **1r4r3r2r** |  |
| **111** | **L** | **L** | **L** | **L** | **L** | **L** | **R** |
| **112** | **H** | **H** | **L** | **L** | **L** | **L** |  |
| **113** | **L** | **L** | **H** | **H** | **L** | **L** |  |
| **114** | **L** | **L** | **L** | **L** | **H** | **H** |  |
| **121** | **L** | **L** | **L** | **L** | **L** | **L** | **T** |
| **122** | **H** | **H** | **L** | **L** | **L** | **L** |  |
| **123** | **L** | **L** | **H** | **H** | **L** | **L** |  |
| **124** | **L** | **L** | **L** | **L** | **H** | **H** |  |
| **131** | **L** | **L** | **L** | **L** | **L** | **L** | **T** |
| **132** | **H** | **H** | **L** | **L** | **L** | **L** |  |
| **133** | **L** | **L** | **H** | **H** | **L** | **L** |  |
| **134** | **L** | **L** | **L** | **L** | **H** | **H** |  |
| **141** | **L** | **L** | **L** | **L** | **L** | **L** | **T** |
| **142** | **H** | **H** | **L** | **L** | **L** | **L** |  |
| **143** | **L** | **L** | **H** | **H** | **L** | **L** |  |
| **144** | **L** | **L** | **L** | **L** | **H** | **H** |  |
| **211** | **L** | **L** | **L** | **H** | **L** | **H** |  |
| **212** | **L** | **L** | **L** | **L** | **L** | **L** | **T** |
| **213** | **H** | **L** | **L** | **L** | **H** | **L** |  |
| **214** | **L** | **H** | **H** | **L** | **L** | **L** |  |
| **221** | **L** | **L** | **L** | **H** | **L** | **H** |  |
| **222** | **L** | **L** | **L** | **L** | **L** | **L** | **R** |
| **223** | **H** | **L** | **L** | **L** | **H** | **L** |  |
| **224** | **L** | **H** | **H** | **L** | **L** | **L** |  |
| **231** | **L** | **L** | **L** | **H** | **L** | **H** |  |
| **232** | **L** | **L** | **L** | **L** | **L** | **L** | **T** |
| **233** | **H** | **L** | **L** | **L** | **H** | **L** |  |
| **234** | **L** | **H** | **H** | **L** | **L** | **L** |  |
| **241** | **L** | **L** | **L** | **H** | **L** | **H** |  |
| **242** | **L** | **L** | **L** | **L** | **L** | **L** | **T** |
| **243** | **H** | **L** | **L** | **L** | **H** | **L** |  |
| **244** | **L** | **H** | **H** | **L** | **L** | **L** |  |
| **311** | **L** | **H** | **L** | **L** | **H** | **L** |  |
| **312** | **L** | **L** | **H** | **L** | **L** | **H** |  |
| **313** | **L** | **L** | **L** | **L** | **L** | **L** | **T** |
| **314** | **H** | **L** | **L** | **H** | **L** | **L** |  |
| **321** | **L** | **H** | **L** | **L** | **H** | **L** |  |
| **322** | **L** | **L** | **H** | **L** | **L** | **H** |  |
| **323** | **L** | **L** | **L** | **L** | **L** | **L** | **T** |
| **324** | **H** | **L** | **L** | **H** | **L** | **L** |  |
| **331** | **L** | **H** | **L** | **L** | **H** | **L** |  |
| **332** | **L** | **L** | **H** | **L** | **L** | **H** |  |
| **333** | **L** | **L** | **L** | **L** | **L** | **L** | **R** |
| **334** | **H** | **L** | **L** | **H** | **L** | **L** |  |
| **341** | **L** | **H** | **L** | **L** | **H** | **L** |  |
| **342** | **L** | **L** | **H** | **L** | **L** | **H** |  |
| **343** | **L** | **L** | **L** | **L** | **L** | **L** | **T** |
| **344** | **H** | **L** | **L** | **H** | **L** | **L** |  |
| **411** | **H** | **L** | **H** | **L** | **L** | **L** |  |
| **412** | **L** | **L** | **L** | **H** | **H** | **L** |  |
| **413** | **L** | **H** | **L** | **L** | **L** | **H** |  |
| **414** | **L** | **L** | **L** | **L** | **L** | **L** | **T** |
| **421** | **H** | **L** | **H** | **L** | **L** | **L** |  |
| **422** | **L** | **L** | **L** | **H** | **H** | **L** |  |
| **423** | **L** | **H** | **L** | **L** | **L** | **H** |  |
| **424** | **L** | **L** | **L** | **L** | **L** | **L** | **T** |
| **431** | **H** | **L** | **H** | **L** | **L** | **L** |  |
| **432** | **L** | **L** | **L** | **H** | **H** | **L** |  |
| **433** | **L** | **H** | **L** | **L** | **L** | **H** |  |
| **434** | **L** | **L** | **L** | **L** | **L** | **L** | **T** |
| **441** | **H** | **L** | **H** | **L** | **L** | **L** |  |
| **442** | **L** | **L** | **L** | **H** | **H** | **L** |  |
| **443** | **L** | **H** | **L** | **L** | **L** | **H** |  |
| **444** | **L** | **L** | **L** | **L** | **L** | **L** | **R** |
