# Supplementary material for: Physical Activity Is Associated with Reduced Implicit Learning but Enhanced Relational Memory and Executive Functioning in Young Adults
Source: PLoS One. 2016 Sep 1;11(9):e0162100. doi: 10.1371/journal.pone.0162100 (PMC5008769; doi:10.1371/journal.pone.0162100)
Supplement: S2 Table — Effects reported in the paper are specific to the first session. (DOCX) [file pone.0162100.s004.docx]

| ***S2 Table*. Relationship between PA*, the three measures comprising PA* (average steps, hours of PA, and number of bouts), and implicit learning collapsed across task sessions**. Effects reported in the paper are specific to the first session. | |
| --- | --- |
|  | Implicit Learning  (collapsed across sessions) |
| PA* | -.25 |
| PA* x Gender | -.28 |
| Avg Steps | -4.2E-6 |
| Avg Steps x Gender | -8.9E-6 |
| Hrs PA | -.02 |
| Hrs PA x Gender | -.01 |
| Bouts | -.01 |
| Bouts x Gender | -.01 |

Beta values reported. Bolded values marked with an asterisk denote significant effects at p<.05, and bolded values without an asterisk denote marginal effects. Age and gender were included as covariates in all models.
